# The Influence of Acid Casein on the Selected Properties of Lime–Metakaolin Mortars

**DOI:** 10.3390/ma16217050

**Published:** 2023-11-06

**Authors:** Przemysław Brzyski, Renata Boris

**Affiliations:** 1 Faculty of Civil Engineering and Architecture, Lublin University of Technology, 40 Nadbystrzycka St., 20-618 Lublin, Poland; 2 Institute of Building Materials, Faculty of Civil Engineering, Vilnius Gediminas Technical University, 28 Linkmenu St., LT-08217 Vilnius, Lithuania; renata.boris@vilniustech.lt

**Keywords:** lime, metakaolin, casein, mechanical parameters, consistency, shrinkage, water absorptivity, mortar

## Abstract

One of the ways to modify selected parameters of lime mortars is the use of biopolymers of animal origin, such as bone glue, skin glue, bovine blood, eggs, and casein. These are protein-based biopolymers. Casein is an example of an organic polymer produced from cow’s milk. The aim of the work was to investigate the possibilities of improving selected properties of mortars based on hydrated lime and metakaolin. The mixture was modified with powdered technical casein in amounts of 0.5%, 1.5%, 1%, 1.5%, and 2% as a partial mass replacement for the binding mixture. Additionally, the influence of increasing the amount of water on the properties of the mortar with a casein admixture of 2% was checked. This study examined consistency, shrinkage, water absorption, capillary action, porosity, flexural, compressive strength, and Young’s modulus. The admixture of casein influenced the properties of the mortar, but not in all cases, and it was possible to determine a clear trend related to the variable amount of casein. Strength properties deteriorated as the amount of casein increased. When air bubbles were introduced into the mortar after the casein was dissolved, the porosity increased as the amount of admixture increased. The moisture properties improved; namely, casein led to a reduction in water absorption and water absorption caused by capillary action. No relationship was observed between the amount of casein and the drying shrinkage. Increasing the amount of water in the mixture led to the expected effects, i.e., an increase in porosity, shrinkage, and water absorption, and a decrease in mechanical strength.

## 1. Introduction

Air lime has been used in construction since ancient times. It was used as a mortar binder to connect masonry elements and as a binder for plasters and paints [1,2]. In ancient Rome, lime was mixed with volcanic dust to obtain a very durable binder [2,3]. Nowadays, in addition to its applications, it is used as a binder in hemp shive-based composites, serving as a thermal insulation wall material [4,5]. It is also used in the production of masonry materials, such as silicate blocks and autoclaved aerated concrete. This work focuses on the use of lime in mortars.

Lime mortars based on air lime are more susceptible to drying shrinkage than mortars based on hydraulic binders or mortars with the addition of pozzolans [6]. Pozo-Antonio J. showed [7] that the shrinkage of air lime mortars is approximately 4.5–7 mm/m, while hydraulic lime mortars shrink at a maximum of 1.5 mm/m. This is due to the slow setting and hardening process, the absence of hydraulic reactions, and the evaporation of a large amount of water introduced into the mortar mixture. One way to prevent shrinkage cracks is to increase the amount of sand [7] and add some kind of fiber. Nayak et al. [8] found that the addition of jute and polypropylene fibers in the amount of 1% and 2% of the cement weight reduced the shrinkage of the cement–lime mortar.

Mortars based only on hydrated lime are characterized by low mechanical strength. Their compressive strength after 28 and 90 days of maturation, depending on the ratio of lime to sand and the ratio of water to lime, ranges from 0.3 MPa to 1.6 MPa [1,7,9,10]. As a result of the secondary carbonation process, the strength increases over time. Silva et al. [11] showed that the strength of a lime mortar exposed to a 3-year period of natural carbonation increased by approximately 25% compared to a 90-day mortar. However, full carbonation of the masonry mortar is practically impossible because of the difficult access of carbon dioxide to the deeper parts of the joint in the masonry. The transformation of calcium hydroxide into calcium carbonate is accompanied by an increase in the volume of the mortar matrix, which narrows the pores and decreases open porosity, hindering the diffusion of carbon dioxide into the mortar [11]. Furthermore, the effectiveness of carbonation is influenced by the ambient temperature and humidity [12]. One way to improve strength parameters, accelerate setting, and increase resistance to water is to partially replace lime with a pozzolanic material. Examples of pozzolans include metakaolin, microsilica, fly ash, volcanic ash, and zeolite. They are characterized by a high content of active silicon oxides and aluminum oxides, which in the presence of water react with calcium hydroxide to create hydrated calcium silicates and aluminates with hydraulic properties. Research [13] showed that the addition of 10% metakaolin increased the compressive strength of a lime mortar by almost seven times, while the same amount of microsilica added resulted in a four-fold increase in compressive strength. In turn, in another study [14], using metakaolin as a lime substitute at an amount of 20%, a fourteen-time increase in compressive strength and a seven-time increase in bending strength were observed. Kang et al. [15] proved that replacing lime with 10% silica fume resulted in an increase in compressive strength after 90 days by approximately 40% and an increase in bending strength by approximately 10%. Pozzolanic materials also influence other parameters of mortars. A study [16] showed that as the content of metakaolin in the mortar increased, the share of pores < 0.1 µm increased. In turn, a 10% lime substitute in the form of silica fume resulted in a reduction in pores with a diameter in the range of 100–200 nm as a result of the pore-filling effect [15]. The partial replacement of hydrated lime with silica fume (10–30%) due to the larger specific surface area and, therefore, the water demand of the pozzolana, resulted in a thickening of the consistency of the fresh mortar [13].

Lime mortars are characterized by higher porosity than, for example, cement mortars or mortars based on hydraulic limes. A lime is a binder with a large specific surface area, but it varies depending on the class of lime, the origin of the limestone, and the method of production. Studies [17,18] showed Blaine fineness values between 7745 cm^2^/g and 16,198 cm^2^/g. Due to the high specific surface of lime, the mortars require larger amounts of mixing water compared to the mortars mentioned above. As a result of binding and hardening through crystallization and carbonation, a significant part of the water is evaporated or absorbed by the masonry substrate, and in its place, a network of pores is created, mainly capillary ones with diameters between 0.1 µm and 100 µm [19,20]. A study [20] showed that the porosity of a mortar based on a hydrated lime was in the range of 20.4–23.1%, depending on the proportion of lime to sand, and as the proportion of sand decreased, the porosity increased. According to research [1], the open porosity of mortars based on air lime with a volume ratio of lime to sand of 1:3 and 1:4 ranged from 27% to 35%. For comparison, the porosity of a mortar based on hydrated lime and pozzolana in a 1:1 ratio was, according to research [21], 16.1 to 19.4%, and increased with an increasing water-to-binder ratio.

Lime mortars are characterized by a greater ability to absorb water compared to mortars based on hydraulic and pozzolanic components. A study [22] showed that the addition of cement to a lime mortar resulted in a reduction in water absorption with increasing cement content (from 2% to 10%). A 10% addition reduced water absorption by almost 19% compared to the reference mortar. Brzyski showed [13] that by replacing 10–30% of hydrated lime with metakaolin, it is possible to reduce the absorbability of mortars by 2.6–9.2%.

One of the groups of admixtures that modify the parameters of lime mortars is organic admixtures of animal origin. Padovnik and Bokan-Bosiljkov [23] showed that the addition of fish animal glue in the amount of 0.34% increased the total and capillary porosity of the lime binder by introducing air bubbles into the mixture during mixing. In another study [24], it was found that as the content of the animal glue admixture increases, the porosity of the lime plaster and the number of pores with a diameter greater than 2 µm increase. Ventola et al. [25] proved that the 5% animal glue admixture improved the compressive strength of a lime mortar by 92% and also reduced the pore volume by approximately 38% and the average pore diameter by approximately 16%. Among the protein admixtures of animal origin, bovine blood was also used in the tests. However, a study was performed that [26] concerned cement mortars. In this investigation, Jasiczak and Zielinski showed that the admixture in the amount of 0.5%, 1%, and 2% in relation to the volume of cement improved the resistance to frost and reduced the strength due to the introduction of air bubbles into the mortar. Chicken egg protein was also used to modify the properties of lime mortars [27,28]. The authors proved that the addition of an egg white in an amount of 2% to 6% improved the workability and strength parameters of the lime mortar.

One of the possible additives that react with lime and affect the properties of mortars and other composites is acid casein. This biopolymer is produced by coagulating casein from skimmed cow’s milk under the influence of acid. Casein is slightly soluble in water but dissolves well in an an alkaline solution, e.g., calcium hydroxide, creating a substance with adhesive properties. Acid casein is technically used as a component of wood and furniture adhesives [29], waterborne coatings [30], and self-leveling concrete [31]. Ventola et al. [25] showed that with the addition of casein in an amount of 5%, the compressive strength after 28 days of lime mortar maturation increased by 68%. In a study [25], it was shown that the 5% casein admixture reduced the pore volume by approximately 17% and also reduced the average pore diameter by approximately 18% compared to the reference lime mortar. Brzyski et al. [32] examined the effect of the casein admixture in an amount of 0.5–5% on the strength and pore distribution in lime–pozzolanic paste. An increase in bending strength and a decrease in compressive strength were demonstrated after the addition of casein to the leaven. The 0.5% admixture increased the strength by approximately 205%, while the 3% admixture reduced the compressive strength by 28%. With an increase in casein content, an increase in porosity and an increase in the average pore diameter were observed. Casein mixed with an alkaline solution was also used as an additive to cemented sand [33]. It was shown that the compressive strength increased with the increase in casein content.

As mentioned in the previous paragraphs, there are several papers describing research on the modification of mortars with organic admixtures of animal origin. However, in individual works, the influence of the admixture varies. This article will help to broaden the knowledge about its influence on selected properties of lime mortars. The aim of this work was to determine the effect of an acid casein and its variable amount on the lime–metakaolin mortar, including the consistency, drying shrinkage, water absorptivity, and capillary rise, as well as the mechanical parameters (flexural and compressive strength) of the samples. However, improving some parameters of lime mortars after modification with casein could broaden the area of application of this material. Taking into account the hydrophobizing effect of casein in the research [34], one of the main goals of this work is to reduce water absorption and capillary rise by adding casein. Any level of improvement in these parameters would be satisfactory, as such results are lacking in the literature. A study found that [32] the flexural strength of lime–pozzolanic paste improved with a 0.5–5% casein admixture. A study showed [25] that the compressive strength of a lime mortar with a 5% casein admixture improved. This work is also an attempt to improve the mechanical properties of a lime mortar using casein in different amounts. This work is a continuation and extension of the authors’ research [32].

## 2. Materials and Methods

### 2.1. Mix Design

Hydrated lime CL-90s was used as the main component of the binder at an amount of 90% of the total weight of the binder. In its chemical composition, lime contains min. 90% CaO + MgO, max. 5% MgO, max. 4% CO_2_, and max. 2% SO_3_. Metakaolinite was used as a partial substitute for lime at an amount of 10% by weight. Metakaolin is a highly reactive pozzolan with a specific surface area of 20,000 cm^2^/g and a specific density of approximately 2.55 g/cm^3^. The main oxides in the composition of metakaolin are SiO_2_ (48–58%), Al_2_O_3_ (37–45%), Fe_2_O_3_ (0.7–2.3%), and TiO_2_ (0.8–2.4%). The water-to-binder ratio was assumed to be 1.0 and is constant for all recipes. Quartz sand with a fraction of 0–2 mm was used as the aggregate. The acid casein admixture was 0.5%, 1%, 1.5%, and 2% by weight as a partial replacement for the lime–metakaolin mixture. The research used acidic casein with a 60-mesh granulation that was unprocessed and purified. It contains protein at an amount of min. 90% (calculated on a dry basis) with a pH in the range of 4.5 to 5.8, and humidity does not exceed 10%. It is a white/cream powder with a typical milky smell. Casein is classified as a phosphoprotein because in its elemental composition, in addition to carbon (53%), hydrogen (7%), oxygen (22%), nitrogen (15.65%), and sulfur (0.76%), it also contains phosphorus (0.85%). Casein was also used in other studies concerning lime–pozzolanic paste [32].

The symbol of the recipe and its composition are presented in Table 1. The number in the lime–metakaolin mortar symbol (LM) represents the percentage of casein (C) in relation to the binder weight (hydrated lime + metakaolin). Furthermore, to investigate the influence of water content on the effectiveness of casein use, two recipes were developed, which contain casein at a 2% amount and variable water/binder ratios (1.05 and 1.10).

### 2.2. Preparation of Specimens

Mortar mixtures were prepared as follows. First, lime, metakaolin, casein, and sand were mixed dry. The water was slowly poured into the resulting mixture and mixed with a mortar mixer. The total mixing time after adding water was 90 s. In [32], when 3% and 5% casein were dissolved in a lime–pozzolanic paste, the mixture liquefied because the air bubbles were introduced into the mixture. In this study, this phenomenon was not observed in the case of mortars. The amount of admixture (max. 2%) was probably too small to achieve a liquefaction effect.

The ready mixture was placed in molds with dimensions of 40 mm × 40 mm × 160 mm in two layers, and each of them was compacted for 15 s on a vibrating table. The samples were aged in air-dry conditions (temperature: 21 °C ± 2 °C and relative humidity: 50% ± 5%) for 28 days. All studies were performed after this maturation period.

### 2.3. Mortar Testing

In this work, the principal properties of the mortar were defined on the base of the standards. The consistency of the fresh mortar had commenced by two methods:

- By flow table according to the PN-EN 1015-3 standard [35];
- By penetration of the plunger according to the PN-EN 1015-4 standard [36].

Three samples for compositions were tested for density, shrinkage, water absorptivity, capillary rise, and flexural strength, and six samples for compositions were tested for compressive strength. The apparent density and the specific density tests were carried out according to the procedures described in the PN-EN 1015-6 [37] and PN-EN 1936 [38] standards. Based on these two parameters, the total porosity was calculated.

The susceptibility of mortars to shrinkage (*Sh_i_*) was determined by measuring the length of the samples immediately after forming (*l*_0_) and after drying to an air-dry state (*l_i_*) Equation (1).

(1) $${Sh}_{i}=\frac{l_{0}-l_{i}}{l_{0}}\cdot 100\%$$

The water absorptivity and the capillary rise tests were carried out according to the PN-EN 13755 [39] and PN-EN 1015-18 [40] standards.

The flexural and compressive strength were measured using an MTS 809 hydraulic press (MTS System Corporation, Eden Prairie, MN, USA) according to the PN-EN 1015-11 [41] standard.

Young’s modulus of the mortars was determined from the slope of the curve in the initial phase, in which the material behaved elastically. The average Young’s modulus was calculated as the ratio of stress change (∆σ) and strain change (∆ε) in this zone of the graph [35]. The stress–strain relationship obtained from the compressive strength test was used for the calculations. Linear regression in the initial zone of the stress–strain diagram allows the determination of Young’s modulus [42,43,44,45].

## 3. Results

### 3.1. Consistency

**Figure 1:**
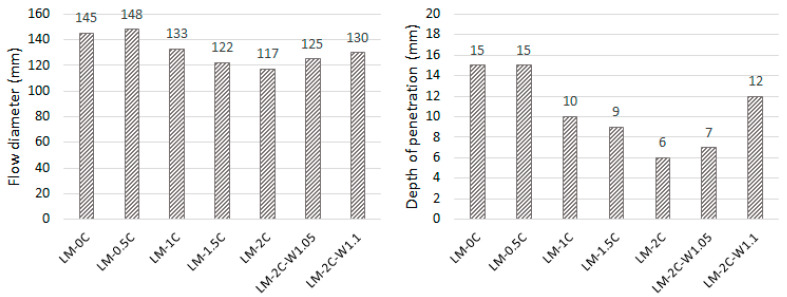
Consistency results tested using the flow table method (**left**) and the plunger penetration method (**right**).

Consistency results tested using the flow table method and the plunger penetration method are shown in Figure 1.

### 3.2. Apparent Density, Specific Density, and Total Porosity

The average values of the apparent density, specific density, and total porosity of the mortars tested are shown in Table 2.

### 3.3. Shrinkage

**Figure 2:**
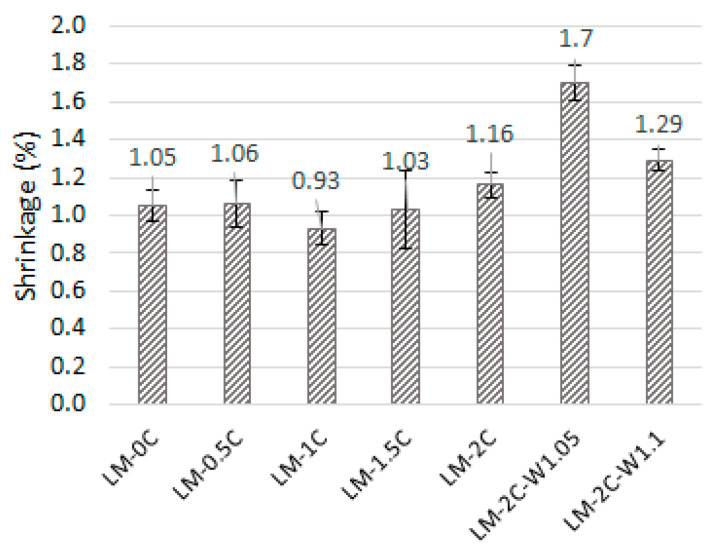
The average decrease in the length of the sample as a result of shrinkage.

The average values of the length loss of the samples as a result of shrinkage are shown in Figure 2. The error bars mean standard deviation.

### 3.4. Mass Absorptivity

**Figure 3:**
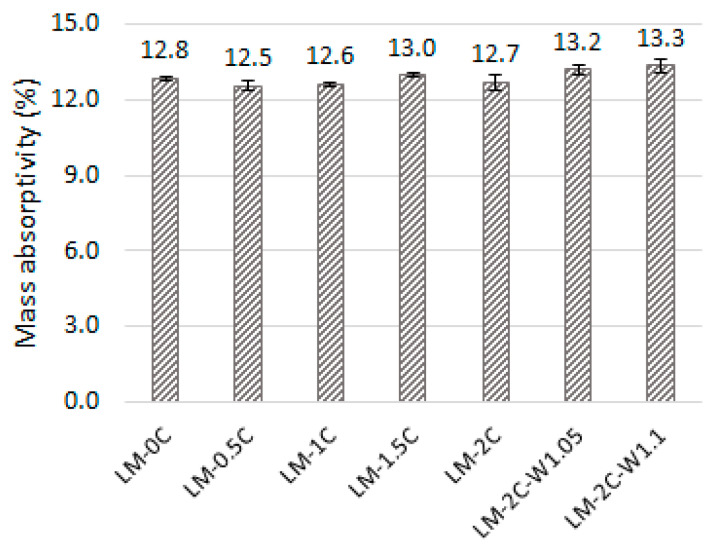
Average values of mass absorptivity of the mortars tested.

The average values of mass absorptivity (water absorption) of the mortars tested are shown in Figure 3. The error bars mean standard deviation.

### 3.5. Capillary Rise

**Figure 4:**
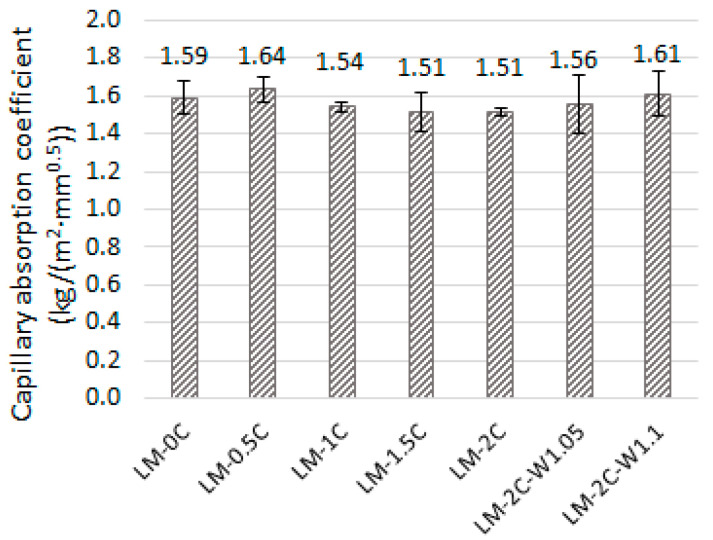
Average values of the capillary absorption coefficient of the mortars tested.

The average values of the capillary absorption coefficient of the mortars tested are shown in Figure 4. The error bars mean standard deviation.

### 3.6. Flexural and Compressive Strengths and Young’s Modulus

**Figure 5:**
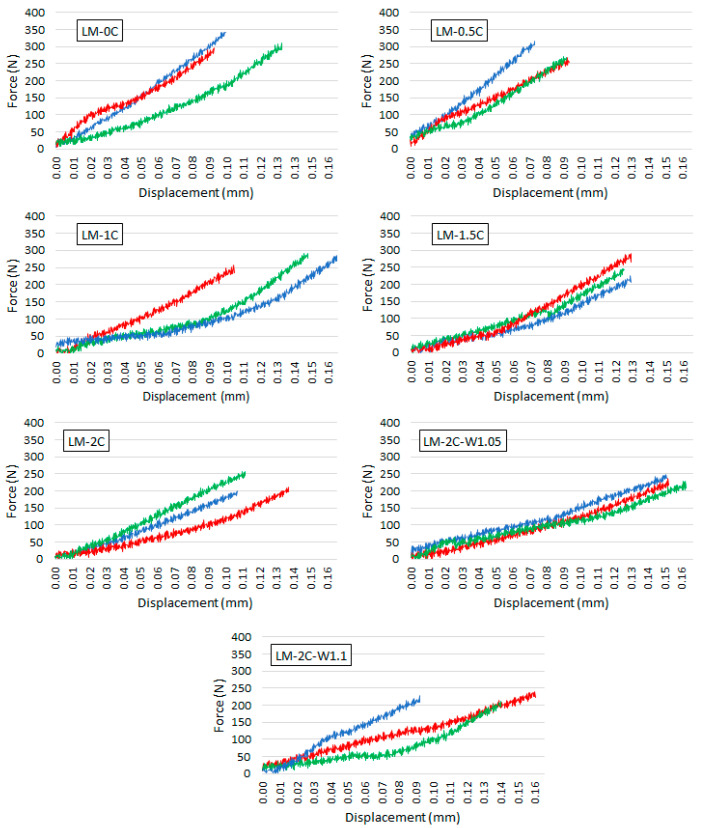
The dependence of the bending force on the displacement of the press head. Each graph shows the results of 3 samples from a given recipe.

The relationship between the bending force and the displacement of the press head of all samples within an individual recipe is shown in Figure 5.

**Figure 6:**
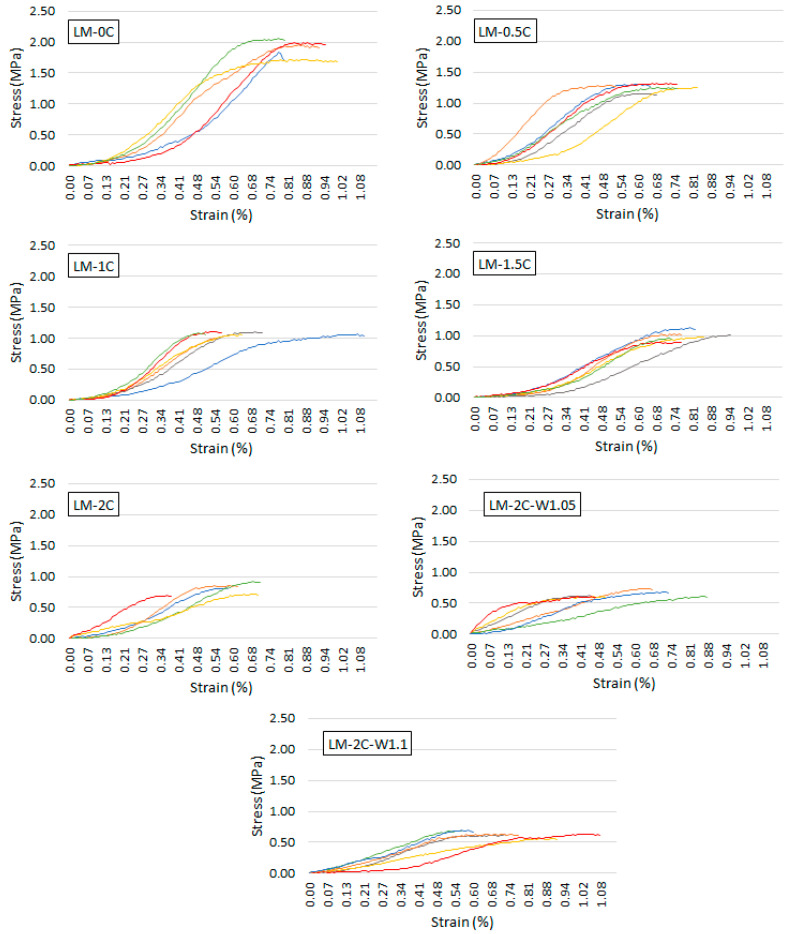
Stress–strain relationship as a result of the compressive strength test. Each graph shows the results of 6 samples from a given recipe.

The relationship between the stress and strain of all samples within an individual recipe as a result of the compressive strength test is shown in Figure 6.

**Figure 7:**
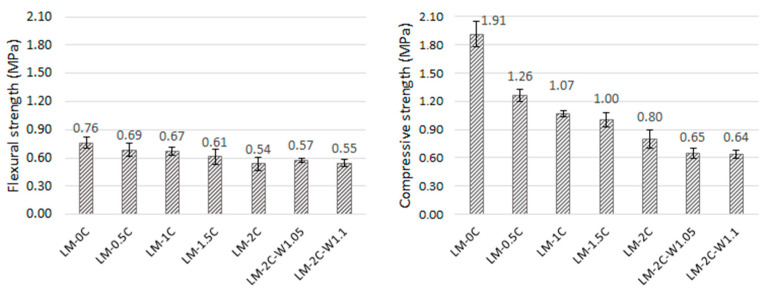
Average values of flexural strength (on the **left**) and compressive strength (on the **right**) of the tested samples (error bars mean standard deviation).

The average values of the flexural and compressive strengths are shown in Figure 7.

**Figure 8:**
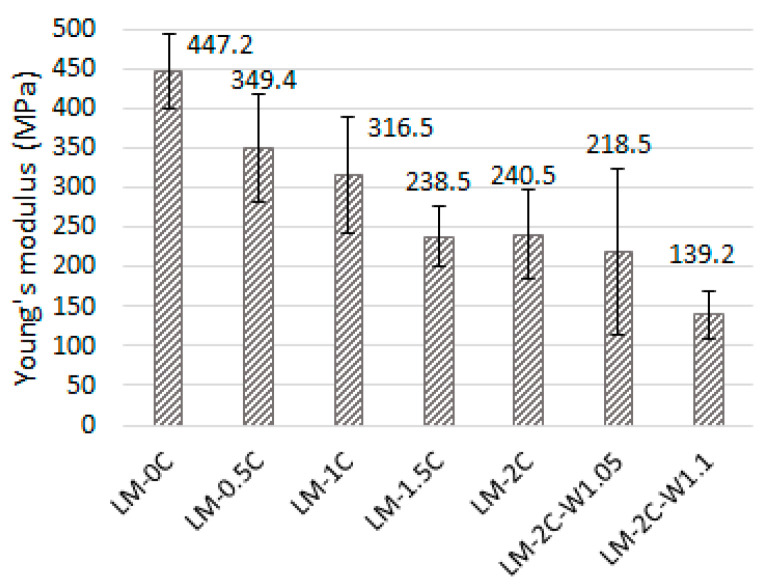
Young’s modulus of the tested mortars.

The results of Young’s modulus are presented in the diagram (Figure 8). The error bars mean standard deviation.

## 4. Discussion

### 4.1. Consistency

Based on Figure 1, it can be concluded that with the addition of casein in the amount of 0.5%, the flow diameter increased, but with increasing admixture content, the fluidity of the mortar deteriorated significantly. The flow diameter and penetration depth are reduced. In [32], the influence of casein in the amount of 0.5%, 1%, 3%, and 5% in the mixture on the properties of lime–pozzolanic paste was examined. It was proven that when 3% and 5% casein were used, the mixture liquefied by generating a large number of bubbles during mixing and, therefore, dissolving the casein in the alkaline leaven. In this work, this phenomenon was not noticed at a casein amount of 2%; therefore, the limit of transition to the liquefied state is in the range of the admixture amount between 2% and 3%. With an admixture of 2%, the flow diameter was reduced by 19.3% and the depth by 60% compared to the reference mortar. In turn, in the case of clay mortar, the addition of casein in the amount of 1.5% (of clay mass) dissolved in the hydrated lime solution caused the mixture to liquefy [34]. Increasing the amount of water from 1.0 to 1.05 and 1.1 also did not result in the more effective dissolution of casein during mixing because it did not cause the mortar mixture to liquefy. The increase in the W/S ratio increased the flow diameter and penetration depth compared to the LM-2.0C recipe, but these values were lower than in the case of the reference mortar.

### 4.2. Apparent Density, Specific Density, and Total Porosity

The apparent density of mortars ranges from 1791 kg/m^3^ to 1856 kg/m^3^. It can be seen that the addition of casein in the amount of 0.5% resulted in a minimal decrease in apparent density. This could be due to technological reasons arising during compaction with technological pores. By increasing the amount of casein to 1% of the weight of the binder, an apparent density was obtained at a level comparable to that of the reference recipe. As the admixture increased, the density decreased, and this phenomenon continued to occur when the amount of water increased. The lowest density was found in the LM-2C-W1.1 recipe, which was approximately 3.5% lower than the reference sample. Increasing the amount of casein in the mixture resulted in an increase in the content of air bubbles. Research [32] confirmed that with an increase in casein content during its dissolution in lime paste and intensive mixing, the intensity of bubble formation increased, and the mixture liquefied. In turn, increasing the amount of water in the composites leads to an increase in porosity due to the evaporation of excess water, resulting in a reduction in apparent density [21,46,47].

### 4.3. Shrinkage

The loss of mortar length ranges from 0.93% to 1.70%. Based on the results obtained (Figure 2), it can be concluded that the water content in the mixture plays a key role in the phenomenon of shrinkage. Increasing the amount of water resulted in an increase in shrinkage of approximately 46% and 11%. However, to determine the dependence of shrinkage on the amount of water, the water dosing range should be increased because the mortar with the highest amount of water (W/S = 1.1) showed less shrinkage than the mortar with the W/S = 1.05 ratio. A slight decrease in the shrinkage value is noticeable in the case of the recipe with 1% casein added compared to the reference mortar; however, due to discrepancies in individual results and slight differences between samples with the same W/S ratio, it is not possible to clearly indicate the effect on shrinkage depending on the amount of added casein. Small differences in shrinkage also result from the same ratio of binder to sand in all mortars because, as shown in other works [7,48], the value of shrinkage increases with an increase in the proportion of sand in the mortar. The shrinkage value for the tested mortars is low due to the use of pozzolana and the faster setting process. In [48], a mixture of air lime and metakaolin (90% + 10%) was also used, and the shrinkage value was comparable (approximately 1.3–1.4%). These values are comparable for mortars based on hydraulic lime, while mortars based on pure lime are characterized by a higher shrinkage in the range of 5–7% [7].

### 4.4. Mass Absorptivity

**Figure 9:**
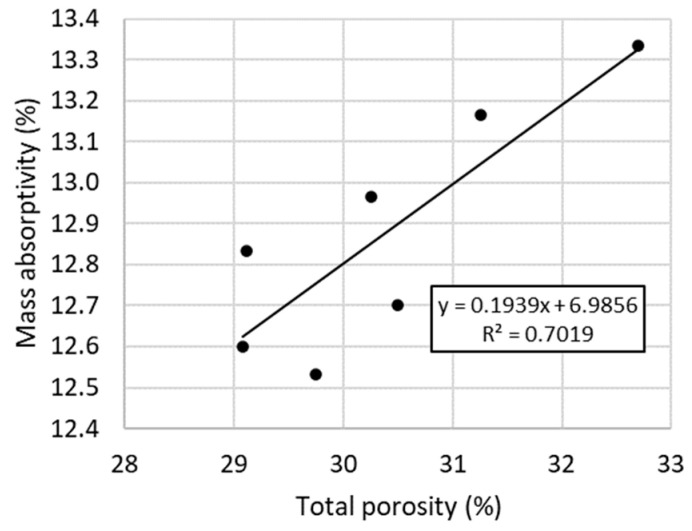
Relationship between mass absorptivity and total porosity of the tested mortars.

The mass absorbability of the mortars ranged from 12.5% to 13.3% (Figure 3). Similar values were shown in tests [49], where mortars based on aerial lime and lime putty were characterized by a water absorption of 10–13.7%. This study showed that the 0.5% casein in the amount of 0.5% caused the greatest decrease in absorbability. As the admixture content increased, the average water absorption of the samples increased. In the case of the LM-2C recipe, there is a noticeable decrease in absorbability despite the increase in casein content, but these results are characterized by the highest standard deviation. As the W/S ratio increased with the same amount of admixture, water absorption increased by 3.9% and 4.7%. The experimental results obtained are highly influenced by the porosity of the materials, which is confirmed by a similar pattern of relationships in this study to determine the total porosity of the recipes analyzed. The relationship between absorptivity and porosity is shown in Figure 9. The LM-1.5C and LM-2C recipes showed greater porosity than the reference sample, hence the higher water absorption of samples made according to the LM-1.5C recipe and individual samples from the LM-2.0 recipe.

### 4.5. Capillary Rise

Based on the results obtained (Figure 4), it can be noted that each mortar achieved a very similar water absorption result by capillary action, which is in the range of approximately 1.50–1.65 kg/(m^2^∙min^0.5^). These values are in the range shown in other tests [1], where the water absorption coefficient of a lime mortar with a lime sand volume ratio of 1:3 ranged from 1.1 to 2.3 kg/(m^2^∙min^0.5^). The lowest absorption value was observed in recipes with an admixture of 1.5% and 2% casein, and in the LM-2C recipe, the individual results were very similar, while the range of individual samples in the LM-1.5C recipe ranged from 1.4 kg/(m^2^∙min^0.5^) to 1.6 kg/(m^2^∙min^0.5^), which in the context of the range of results of the entire study is a clearly large spread. Despite slight differences between individual recipes, the sample with 0.5% casein showed the least favorable results. Increasing the amount of water in the recipe containing 2% casein resulted in an increase in the capillary action. This is related to the increase in porosity and probably the expansion of the capillary pore network through the evaporation of excess mixing water.

### 4.6. Flexural and Compressive Strength and Young’s Modulus

**Figure 10:**
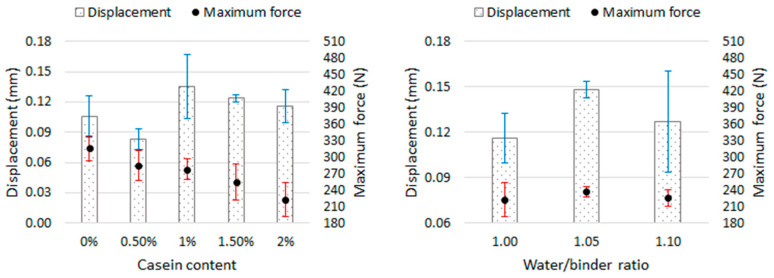
Relationship between casein content, displacement (deflection), and maximum force (on the **left**) and between the water/binder ratio, displacement (deflection), and maximum force (on the **right**).

Based on the force-displacement graphs (Figure 5), it was shown that the deflection of the mortar samples at the destructive force in the flexural strength test ranged from 0.07 mm to 0.16 mm. Figure 10 shows the dependence of the displacement of the press head (meaning the deflection of the sample) and the maximum force on the casein content in the mortar and on the water content in the mortar. Error bars (blue and red color) mean standard deviation.

There is no clear relationship between the susceptibility of mortar samples to deflection and the casein content or water-to-binder ratio. Mortars with a casein content of 1.5–2% were destroyed with a higher deflection than the reference sample by approximately 10% to 28%, despite the lower destructive force. As the casein content increased, the deformation of the samples and the destructive force decreased. Therefore, the presence of casein resulted in a greater deformability of the lime mortar. The exception is the mortar with an admixture of casein in the amount of 0.5% because both the destructive force and the deflection were lower than in the case of the reference mortar. In the case of modifying the mortar LM-2C with the amount of water, the destructive force was at a similar level in the three recipes; however, increasing the W/S ratio to 1.05 resulted in an increase in mortar deformation (deflection) of the mortar by approximately 28%. A further increase in W/S (to 1.1) resulted in a reduction in deflection, but it was still greater than in the case of W/S = 1. However, in the latter case, a large scatter of results was observed.

**Figure 11:**
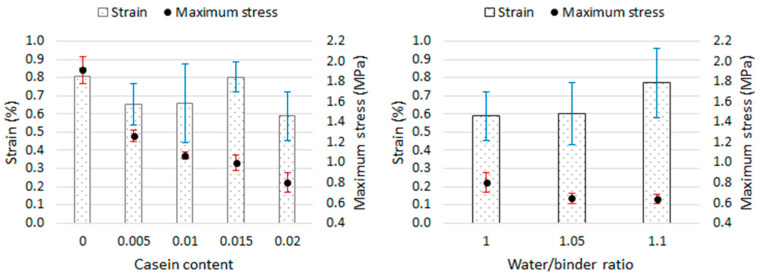
Relationship between casein content, strain, and maximum stress (on the **left**) and between the water/binder ratio, strain, and maximum stress (on the **right**).

Based on the graphs of the stress–strain relationship (Figure 6), it was shown that the stress of the mortar samples at the destructive stress in the compressive strength test ranged from 0.59% to 0.81%. Figure 11 shows the dependence of the strain and the maximum stress on the casein content in the mortar and on the water content in the mortar. Error bars (blue and red color) mean standard deviation.

The flexural strength of mortars ranges from 0.55 MPa to 0.76 MPa, and the compressive strength ranges from 0.64 MPa to 1.91 MPa. In both cases, there is a visible tendency to decrease strength with increasing casein content. The 2% admixture caused a decrease in strength of almost 29% (flexural) and almost 58% (compressive) compared to the reference mortar. In [32], the influence of casein on the strength of lime–pozzolanic paste was investigated. A decrease in strength was also observed with an increase in the share of the admixture, but despite this, in the case of flexural strength, the pastes containing casein were characterized by values higher than those of the reference sample. The author suggested that the formed casein glue could have increased the adhesion of the binder particles, enhancing the strength. In the case of compression, only the paste with a 0.5% admixture of 0.5% showed a higher strength than the reference paste. In this work, sand is an additional ingredient; further research should examine the contact zone between the matrix and the aggregate and whether the entanglement of the lime particles with casein glue has not weakened this zone. It was also shown that with an increase in the content of casein, the porosity of the mortars increased as a result of the formation of air bubbles during the dissolution of casein, which was also confirmed in [32], and in the case of many materials, the strength deteriorates with increasing porosity. In [32], it was also found that the cause of the decrease in shaking with increasing casein content could be the increase in the average diameter of the pores as the amount of admixture increases. Increasing the water-to-binder ratio did not result in a decrease in flexural strength. The values remained at a similar level when taking into account the standard deviation. Thinning the consistency with increasing water content could have a positive effect on the coverage of the aggregate with the paste. In turn, after increasing the water-to-binder ratio from 1.0 to 1.05, the compressive strength decreased by approximately 19%. This procedure also resulted in an increase in shrinkage, which could lead to cracks that formed, weakening the mortar matrix. Many studies have shown that the compressive strength of mortars decreases as the water content increases [50,51,52].

The value of the static modulus of elasticity in the city ranges from 139.2 MPa to 447.2 MPa. As in the case of compressive strength, the modulus value decreases with increasing casein content. Coating the aggregate of the binder particles with casein glue led to an enhanced plastic behavior of the mortars under static load. However, significant standard deviation values can be observed in most recipes. Increasing the W/S ratio also resulted in a decrease in the modulus of elasticity. In [16], lime and matakaolin mortars were shown to have values similar to those selected from this research (300–451 MPa), and it was stated that low modulus values may result from the presence of cracks resulting from shrinkage. This statement was confirmed in our research because the lowest modulus values, namely 218.5 MPa and 139.2 MPa, were demonstrated by mortars with an increased amount of water, and at the same time were characterized by the highest porosity and shrinkage. For comparison, other studies [45] have shown that the modulus of elasticity of a mortar based only on air lime is approximately 70 MPa after 28 days of hardening with a compressive strength of 0.6 MPa, while mortars based on hydraulic lime have a modulus of 130–278 MPa with a strength of 1.9–3.9 MPa.

**Figure 12:**
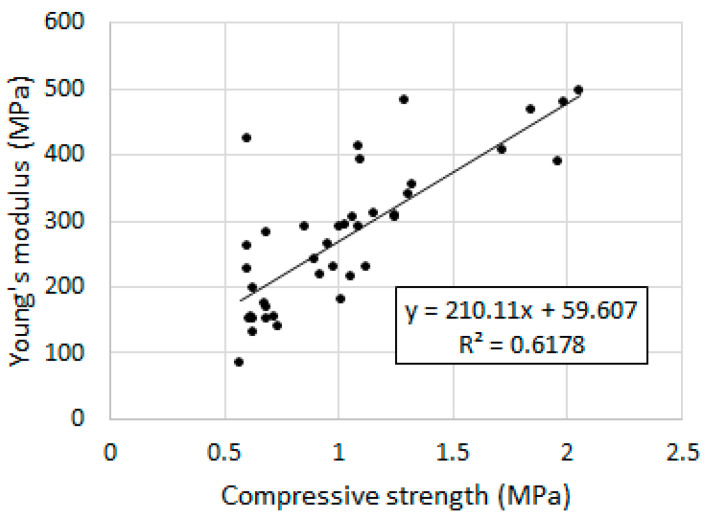
Young’s modulus and compressive strength relationship.

The graph (Figure 12) shows the relationship between Young’s modulus and the compressive strength of the mortars.

Generally, a trend of increasing elastic modulus with increasing compressive strength can be observed. However, in some cases, there are deviations from this trend, as evidenced by the low value of the coefficient of determination R^2^ = 0.6178. Ref. [45] showed the dependence of the compressive strength of a wall based on lime mortars on the elastic modulus of this wall, determined by the coefficient of determination at the level of R^2^ = 0.7766. An increase in the modulus value with an increase in compressive strength was also demonstrated.

## 5. Conclusions

On the basis of the obtained results, it is possible to draw the following conclusions:

- The consistency of mortar mixtures thickens as the casein content increases. Increasing the amount of water in the mortar with 2% casein plasticizes the consistency, but it is still thicker than in the case of the reference mortar.
- The porosity of mortars increased with an increase in the content of a casein admixture. A more dynamic increase in porosity occurred with an increase in the water content.
- There was no dependence of shrinkage on the amount of casein added. The most noticeable increase in shrinkage occurred in a mortar with an admixture of 2%. Increasing the amount of water increased the shrinkage value (by 46% and 11%).
- Selected amounts of casein added (0.5%, 1%, 2%) slightly reduced water absorption. Water absorption increased (by 3.9% and 4.7%) when the water-to-binder ratio increased.
- The addition of casein reduced the capillary rise of water. Only an admixture of 0.5% increased the water absorption capacity.
- As the casein content increased, the flexural strength (by 9–29%) and the compressive strength decreased (by 34–58%). Increasing the w/s ratio did not cause significant changes compared to w/b = 1 in the case of bending strength but reduced compressive strength by 19–20%.
- As the casein content increased, the value of the elastic modulus decreased. Increasing the w/s ratio also resulted in a decrease in the value of Young’s modulus.

The addition of casein did not have a positive effect on the mechanical properties of lime mortars. The goal of improving strength, which was demonstrated in other works, such as [32] and [25], was not achieved. Among the assumed goals, only selected casein-modified mortar recipes showed lower water absorption and lower capillary absorption. However, it should be checked in further tests whether this will also result in reduced vapor permeability. Increasing the amount of water in a mortar containing 2% casein did not improve its parameters, which ruled out the expectation that increasing the w/b ratio would allow a more effective dissolution of casein and thus improve its parameters. Studies on the effect of one casein among many available on the market were presented. It would be necessary to check the influence of the method of sample preparation, the type of casein, and its grinding on the properties of mortars to draw broader conclusions. The results obtained and the causes of changes occurring in individual mortar recipes are not yet sufficiently explained. The authors also intend to investigate the chemical phenomena occurring in the modified mortar and understand the results obtained more deeply and precisely.

## Figures and Tables

**Table 1:** Components of the mortars tested (proportions by mass).

Recipe Symbol		Components		
	Binder	Casein:Binder	(Binder + Casein):Sand	Water/(Binder + Casein)
LM-0C	Hydrated lime 90% Metakaolin 10%	0:1	1:5	1.00
LM-0.5C		0.05:0.995		
LM-1C		0.01:0.99		
LM-1.5C		0.015:0.985		
LM-2C		0.02:0.98		
LM-2C-W1.05		0.02:0.98		1.05
LM-2C-W1.1		0.02:0.98		1.10

**Table 2:** Apparent density, specific density, and total porosity of the mortars.

Parameter	LM-0C	LM-0.5C	LM-1C	LM-1.5C	LM-2C	LM-2C-W1.05	LM-2C-W1.1
Apparent density (kg/m^3^)	1854	1840	1856	1837	1822	1804	1791
Specific density (kg/m^3^)	2616	2619	2619	2634	2623	2624	2661
Total porosity (%)	29.1	29.8	29.1	30.3	30.5	31.3	32.7

## Data Availability

Data are contained within the article.
